# *Lactobacillus plantarum ferment* of *forsythia suspensa* mitigates UVB-induced photoaging in HaCaT keratinocytes by modulating the metabolite profile and enhancing anti-photoaging activities

**DOI:** 10.1186/s40643-025-00964-8

**Published:** 2025-11-05

**Authors:** Yutao He, Yuzhi Zhang, Ning Su, Yunxia Chen, Li Yang, Hao Fu, Dongdong Wang, Changtao Wang, Meng Li

**Affiliations:** 1 Beijing Key Laboratory of Plant Resource Research and Development, School of Light Industry Science and Engineering, Beijing Technology and Business University, Beijing, 100048 People’s Republic of China; 2 State Key Laboratory of Bioreactor Engineering, Department of Food Science and Technology, School of Biotechnology, East China University of Science and Technology, Shanghai, 200237 People’s Republic of China; 3 Institute of Cosmetic Regulatory Science, Beijing Technology and Business University, Beijing, 100048 People’s Republic of China; 4 Chinese Academy of Inspection and Quarantine, Beijing, 100176 People’s Republic of China; 5 Beijing Sino-German Union Cosmetic Institute Co., Ltd, Beijing, 100176 People’s Republic of China

**Keywords:** Forsythia, Ferment, UVB, Antioxidant, Anti-inflammatory

## Abstract

The aqueous extract and *Lactobacillus plantarum* fermentation broth were prepared from forsythia as plant raw material, and the two extracts were investigated for the evaluation of cosmetic efficacy in antioxidant, anti-inflammatory repair, and safety after the establishment of the UVB damage HaCaT cell model. The results showed that the forsythia fermentation broth and the aqueous extract had good scavenging activities against DPPH radicals, hydroxyl radicals, ABTS radicals and reactive oxygen species, and the fermentation broth had significantly stronger antioxidant effects than the aqueous extract. At the level of protein expression and gene transcription, the forsythia fermentation broth reduced the content of inflammatory factors (TNF-α, IL-8, IL-6, IL-1β) and apoptotic factors (Caspase-3, Caspase-9, Bax, Bcl-2) in the cells and even tended to the normal level, and the effect was higher than that of aqueous solution as the blank control, which indicated that the forsythia fermentation broth had anti-inflammatory and restorative effects. Full metabolite profiling by chromatographic tandem mass spectrometry (HPLC–MS/MS) of the aqueous extract and forsythia fermentation broth revealed that the fermentation broth modulated the metabolite profile. This modulation was associated with significantly enhanced protective effects, as evidenced by boosted oxidative stress and anti-inflammatory responses at the cellular level against UVB-induced HaCaT cells. Using chicken embryo allantoic membrane eye irritation experiment and erythrocyte haemolysis experiment on the aqueous extract and forsythia fermentation broth for safety evaluation experiments, the irritation scores were 0.09, 0.08, respectively, and there was no haemolysis of blood vessels, which indicated that the aqueous extract of forsythia and the fermentation solution did not have eye irritation, and that the use of forsythia fermentation broth in a safe range had no detrimental effect on cell membranes and was not irritating.

**Graphical abstract:**

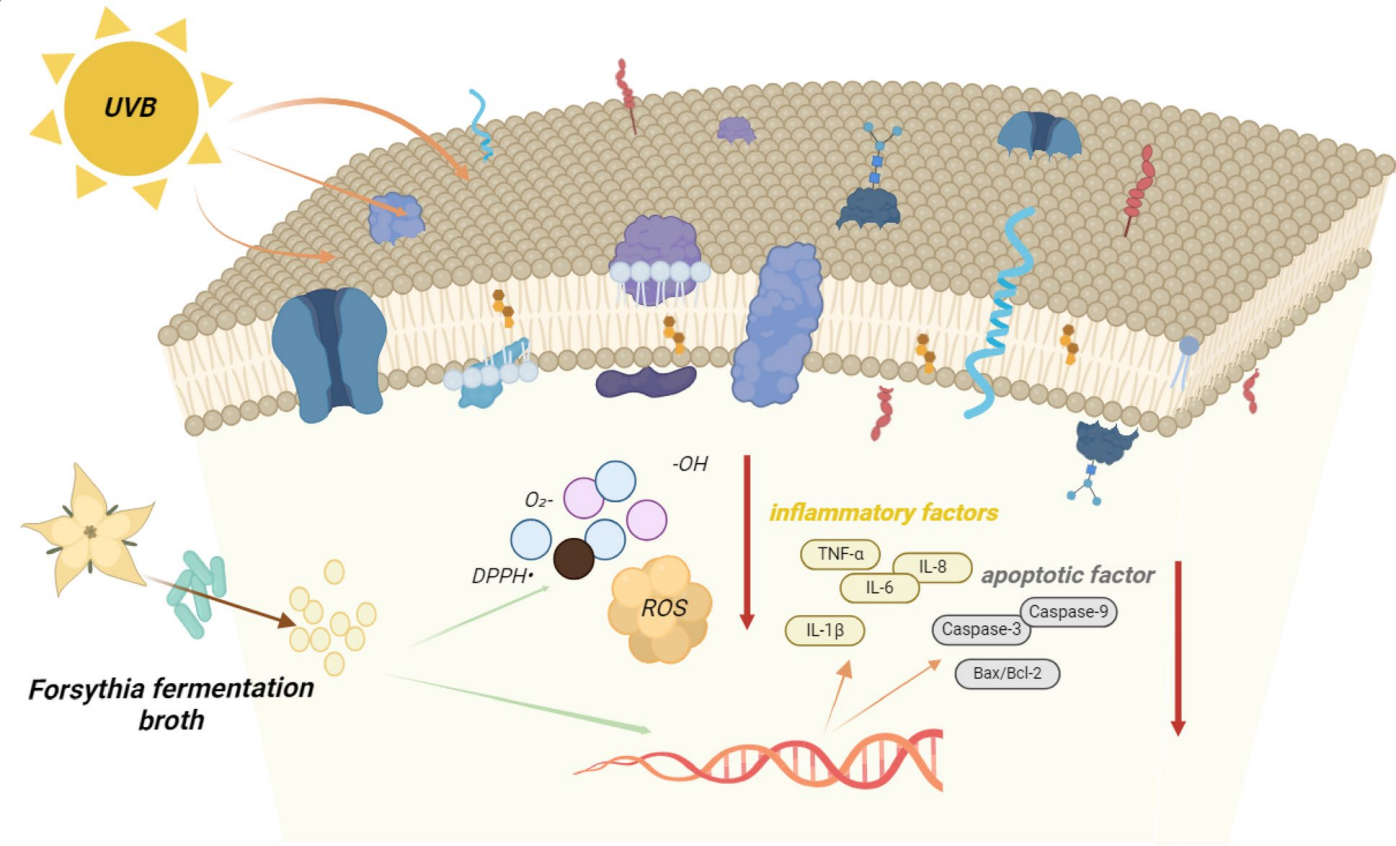

## Introduction

Forsythia (*Forsythia suspensa* (Thunb.) Vahl) is a traditional Chinese medicine in China, a deciduous shrub of the genus Forsytia. It is a deciduous shrub of the genus Forsythia (Luo et al. 2022). The fruit is used as a medicine and is called "forsythia seed" in Chinese medicine, and its shell is a medicinal part of the pharmacopoeia (Dong et al. 2017). In the Chinese Pharmacopoeia, forsythia has the effect of clearing heat and removing toxins, subduing swellings, dispersing wind and dissipating heat (also known as fever and cold) (Peng et al. 2020). From forsythia more than 200 compounds have been isolated and identified so far, and the compounds present are mainly concentrated in phenylethanol glycosides and lignans, followed by C6-C2 natural alcohols and their glycosides, terpenes, volatile oils, flavonoids, phenolic acids, sterols, and alkaloids (Zhao et al. 2022). Forsythia has potential medicinal value in neuroprotection, hepatoprotection and choleretic, immunomodulation, antioxidant action, diuretic and antihypertensive, antiaging, antitumour, antiviral, antibacterial, and anti-inflammatory properties (Wang et al. 2018; Yi et al. 2019; Jeong et al. 2021; Guo and Zhu 2022; Chao et al. 2022; Jin et al. 2023; Liu et al. 2023). Currently, the primary products of forsythia are mainly medicines, forsythia tea, forsythia essential oil, preservatives, etc. Fermented products are mainly used to develop fermented beverages, teas, and medicines for animal use using its anti-inflammatory, antibacterial, and antiviral effects (Yang et al. 2014; Huang 2020; Shen et al. 2021). No research has been found on forsythia fermentation broth in the field of cellular photoaging such as antioxidant, anti-inflammatory, and cellular repair.

Exposure of the skin to external environmental factors such as environmental toxins, pathogens and ultraviolet (UV) radiation also contributes to the external ageing of the skin, accelerating the overall ageing process of the skin. Among the external environmental factors that cause skin aging, UV radiation is the most important factor (Sanches Silveira and Myaki Pedroso 2014). The energy of UVB is high but its wavelength is short, and therefore, it is mainly absorbed by the epidermis (Battie et al. 2014). Keratinocytes make up 90–95% of epidermal cells and are the main target cells of UVB radiation (Tang et al. 2021). The changes that occur in the skin after exposure to UV radiation are related to changes that occur at the cellular level (Guo et al. 2022). Therefore, the present study was conducted to investigate the effect of forsythia fermentation broth on UVB-induced epidermal keratinocytes using UVB-irradiated HaCaT cells as an in vitro model.

A large number of experiments have proved that after fermentation, the content of active ingredients of plant raw materials increased, antioxidant properties were enhanced, anti-inflammatory effect increased, the extraction rate and utilisation rate were higher, and it had a synergistic effect (Bhanja Dey et al. 2016). For example, Zhao et al. (Zhao et al. 2016) found that Ganoderma lucidum fermentation broth with volume fractions of 25. 89% and 42. 18% scavenged 50% of DPPH radicals and hydroxyl radicals, respectively. (Mo et al. 2021) found that Salvia divinorum fermentation broth exhibited a protective effect against hydrogen peroxide induced damage to cells. (Yan et al. 2023) found that Xanthium fermentation broth has anti-inflammatory and antioxidant effects etc. which can be used as efficacious raw material in cosmetics. In a related advancement, regenerated extracellular vesicles from L. plantarum were shown to enhance keratinocyte survival following UVB radiation and delay skin cell aging (Qin et al. 2024).

Based on the above experimental basis, this experiment compares the antioxidant and anti-inflammatory repair efficacy as well as safety of the aqueous extract of Forsythia and Forsythia fermentation broth by establishing a UVB cell damage model and comparing the antioxidant and anti-inflammatory repair efficacy as well as safety of the aqueous extract of Forsythia with that of Forsythia fermentation broth at the cellular level as well as at the levels of protein expression, gene transcription, and stimulation experiments, so that the Forsythia fermentation broth can provide theoretical evidence for the protection of HaCat cells against photo-aging induced by UVB.

## Materials and methods

### Materials

Forsythia, Beijing Tongrentang Co. Ltd; *Lactobacillus plantarum* [CICC 20261], China Industrial Microbial Strain Preservation and Management Centre; Human Epidermal Immortalisation (HaCaT) Cells, China Research Institute of Inspection and Quarantine; Fetal Bovine Serum, Dual Antibody (Penicillin–Streptomycin), DMEM Medium, Gibco, USA; Isopropyl Alcohol, Chloroform, Ethyl Alcohol, SINOTROPICAL GROUP; Sodium Dodecyl Sulphate, NaoH, Nacl, anhydrous ethanol, Sinopharm Chemical Reagent Corporation; Cell Counting Kit-8 (CCK-8) Kit, BCA Protein Concentration Measurement Kit, Reactive Oxygenation Kit, Human TNF-α (Tumour Necrosis Factor α) Elisa Kit, Human IL-1β (Interleukin 1β) Elisa Kit, Human IL-6 (Interleukin 6) Elisa Kit, Human IL-8 (Interleukin 8) Elisa Kit, DEPC water, Beijing Barege Biotechnology Co., Ltd; Ferrous Sulfate, Hydrogen Peroxide, Salicylic Acid, Beijing Chemical Factory; Caspase-3 Activity Detection Kit, Caspase-9 Activity Detection Kit, BCL-2 Associated X Protein (Bax) 2-related X protein (Bax) kit, B-cell lymphoma factor-2 (BCL-2) test kit, Nanjing Jianjian Biotechnology Co. Ltd; chicken embryo, Linhai Xingui Family Farm Co. Ltd; fresh rabbit blood, Xinglong Experimental Animal Breeding Farm, Haidian District, Beijing; phosphate buffer solution (PBS), Shanghai Biyuntian Biotechnology Co. Methanol, acetonitrile, isopropanol, Merck KGaA; Formic acid, Sia reagent; 2-chlorophenylalanine Shanghai Yuanye Biotechnology Co.

Pulveriser, Beijing Xingshili Technology Development Co; Shaker, Shanghai Yiheng Scientific Instrument Company; Constant Temperature Incubator, Fisher Scientific; Ultra-clean Bench, YT-CJ-1ND Ultra-clean Bench, Beijing Yatai Cologne Instrument Technology Company; High-speed Centrifuge, Low-speed Centrifuge, Hunan Xiangyi Instrument Development Co. Ltd.; analysis balance, Beijing Fuhai Science and Technology Co., Ltd.; electric constant temperature blast drying box, Shanghai Yuejin Medical Equipment Co., Ltd.; PCR instrument, Shanghai Tianneng Life Science Co. Ultra High Performance Liquid Chromatograph, Thermo Fisher Scientific; High Resolution Mass Spectrometer, HF Thermo Fisher Scientific; Chromatographic column, Agilent technologies; Centrifuge, Changsha Hi-Tech Industrial Development Zone Xiangyi Centrifuge Instrument Co. Ltd; CNC Ultrasonic Cleaner KQ-00DE Kunshan Ultrasonic Instrument Co., Ltd; Freeze Dryer FD-IA-50 Shanghai Bilang Instrument Manufacturing Co.

### Preparation of forsythia fermentation broth and aqueous extract

Dried fruits of *Forsythia suspensa* (Beijing Tongrentang Co. Ltd) were used as raw material. After the forsythia was dried, it was crushed with a high-speed multifunctional grinder, sieved, and then purified water was added into the conical flasks according to the material-liquid ratio of 1:20, sealed and autoclaved, and then accessed to *Lactobacillus plantarum* strains after cooled down to room temperature and cultivated in a shaker at 28 ℃ and 180 r/min for 48 h, and then centrifuged for 30 min to take the supernatant, which is the forsythia fermentation broth as the samples of the subsequent experiments (FF).

Preparation of forsythia aqueous extract (blank control) (FW)was performed as above, but without inoculation of bacteria.

DMEM culture medium without 10% fetal bovine serum and with 1% penicillin–streptomycin was prepared, and different gradient concentrations of fermentation broth were obtained by diluting the original fermentation broth, which was used for the subsequent cell experiments.

### Cell viability test

The HaCaT cell culture flasks were removed from the incubator and the cell status was observed. The cells were inoculated into 96-well plates and cultured at 37 ℃ in 5% CO_2_ for 12 h. The rest of the steps were performed according to the CCK8 assay kit, add different concentration of samples to each sample group and make 6 parallel groups, and finally the cell viability was calculated based on the absorbance of different samples at 450 nm in order to derive the optimal sample volume fraction.

### HaCaT cell culture

HaCaT cells were removed from liquid nitrogen and placed in a 37 °C water bath for rapid lysis. 5 mL of complete medium (DMEM culture medium containing 10% fetal bovine serum and 1% penicillin–streptomycin) was inhaled in advance in a T25 cell culture flask, and then the cells were placed in the culture flask. Place the culture flasks in a 37 ℃ constant temperature incubator, observe the cell status, and pour out the culture medium when the amount of cell attachment reaches 80%-90%. The cells were washed twice with 2 mL of PBS buffer, 1 mL of trypsin was added for digestion, and 2 mL of culture medium was added. The cells were centrifuged at 2000 r/min for 5 min to enter the next round of passaging culture.

### UVB modelling

HaCaT cells with good growth status at a density of 1.0 × 10^4^ cells/well were added to 96-well plates, and after 12 h of culture, HaCaT cells were irradiated with UVB at different intensities, and PBS was aspirated and discarded before and after, and then cultured for another 12 h. The optimal UVB intensities were obtained by calculating the IC50 values using the CCK8 method.

### Determination of antioxidant efficacy of FF

#### Measurement of free radical scavenging capacity of FF

The scavenging effect of different free radicals such as DPPH radical, hydroxyl radical, and ABTS radical can measure the in vitro antioxidant capacity of the samples, and the samples have different inhibitory effects on different free radicals, and the antioxidant capacity of the samples can be comprehensively evaluated by the scavenging rate of different free radicals. Refer to the instruction manual of the corresponding kit for specific operation.

#### Determination of reactive oxygen content

The HaCaT cell culture flasks were taken out of the incubator and the cell status was observed. Six-well plates were inoculated with 2 mL of HaCaT cell suspension diluted in DMEM complete culture medium per well, and blank control group, model group and sample group were set up. The number of cells per well was controlled to be 500,000. After incubation at 37 ℃ for 12 h in a 5% CO_2_ incubator, the culture medium was discarded. Add 1 mL of PBS to each well. The above cells were irradiated with UVB while the blank group was not irradiated with UVB. The rest of the steps were carried out according to the instructions of the Reactive Oxygen Kit.

### Determination of anti-inflammatory and repairing efficacy

#### cell processing

Remove the HaCaT cell culture flask from the incubator and observe the cell status. The cells were evenly spread in 6-well plates. The incubator conditions were set at 37 ℃, 5% CO_2_, and the cells were cultured for 12 h. The medium was discarded, 1 mLPBS was added, and after irradiation with UVB, PBS was discarded and the samples were cultured for one day. After incubation, the cells were washed twice with PBS, 200 μL of lysate was added to each well, and the cells were scraped off with a spatula in a centrifuge tube after 2 min of resting, and the cell lysate supernatant was obtained by centrifugation at 4 ℃ for 2 min.

#### Measurement of protein expression of cellular inflammatory factors and apoptotic factors

After cell treatment in accordance with 2.7.1, in accordance with the instructions of the kit, the protein content expression of inflammatory factors and apoptotic factors in HaCaT cells was detected by ELISA. The blank group was treated with no sample and no cells; the model group was the cell model after UVB irradiation.

The results of ELISA were calibrated according to the instructions of BCA Protein Concentration Assay Kit.

#### Measurement of relative expression of cellular inflammatory and apoptotic factors

The qPCR was performed according to the TransStart Top Green qPCR SuperMix kit instructions. Gene expression of inflammatory and apoptotic factors was detected in the cells after extraction of total RNA from the sample cells by TRIzol method.

The primers were designed according to the gene sequences published in NCBI, and the primers specific to 9 target genes (including the housekeeping gene β-actin) were designed by Primer Express software, and their sequences are shown in Table 1.

**Table 1:** Primer sequence

Gene	Primer (F, R)
TNF-α	AGTGGTGCCAGCCGATGGGTTGT
	GCTGAGTTGGTCCCCCTTCTCCAG
IL-1β	CATGAGCACCTTCTTTTCCT
	TGTACCAGTTGGGGAACTCT
IL-6	GACAGCCACTCACCTCTTCA
	TTAACCAGGCAAGTCTCCTC
Bcl	GACTTCGCCGAGATGTCCAG
	CGGTGCTTGGCAATTAGTGG
Bax	ATGGAGCTGCAGAGGATT
	AATGTCCAGCCCATGATGGTTC
Caspase-3	GTACAGAGCTGGACTGCGGTATTG
	AGTCGGCCTCCACTGGTATCTTC
Caspase-9	GGTGGACATTGGTTCTGGCAGAG
	ACGTTGTTGATGATGAGGCAGTGG
IL-8	CTGCGCCAACACAGAAATTATTGTA
	TTCACTGGCATCTTCACTGATTCTT
β-actin	CTGAAGCCCCACTCAATCCA
	GCCAAGTCAAGACGGAGGAT

### Full metabolite profiling by HPLC–MS/MS for FF and FW

Take an appropriate amount of well-mixed samples in a 2 mL centrifuge tube; solid samples: add 1 mL of 70% methanol solution and 3 mm specification steel beads, and grind them for 3 min with a fully automated sample fast grinder (JXFSTPRP-48, 70 Hz), remove them, and vortex for 10 min for mixing; liquid samples: add an appropriate amount of pure methanol, and vortex for 10 min for mixing the samples; centrifuge the mixed sample solution at 4℃ and 12000 rpm for 10 min; add 100 μg/mL internal standard solution (2-chlorophenylpropanol) in the supernatant through 0.22 μm microporous filter membrane. Centrifuge at 12000 rpm at 4℃ for 10 min; the supernatant was passed through 0.22 μm microporous membrane, and 100 μg/mL internal standard solution (2-chlorophenylalanine) was added to make the concentration of 1 mg/L, and then detected on the machine.

Based on the metabolite peak area in each sample, the relative concentration of metabolite in the sample was calculated. Accurately measure 500ul of Forsythia aqueous extract and 500ul of Forsythia BC-3, add 500ul of methanol, shake and mix well, centrifuge at 12000r/min for 10 min, and then pass through the membrane on the machine. Based on the metabolite peak area in each sample, the relative percentage of metabolites in the sample was calculated.

The LC–MS instrument was used as the analytical platform, and C18 was used as the chromatographic column at 30 °C; the flow rate was 0.3 mL/min; the mobile phases were composed of A: 0.1% formic acid aqueous solution, and B: pure acetonitrile; the injection volume was 2 μL, and the temperature of the autosampler was 4 °C for chromatography-mass spectrometry (Chromatography-Mass Spectrometry).

### Determination of safety and efficacy of FF

#### Hemolysis of red blood cells

Firstly, the samples were treated by freeze-drying the fermentation broth and diluting different concentrations with PBS solution. Afterwards the red blood cell suspension (RBC) was pre-treated: fresh rabbit blood was diluted 3:7 with PBS solution and shaken well. The supernatant was discarded and PBS buffer was added to 40 mL and repeated two to three times for decontamination.

Take 1 mL of precipitate and add 9 mL of PBS and mix well. RBC was prepared by taking 250 μL and adding 750 μL of ultrapure water to make up to 1 mL, 150 r/min, and incubating in a shaker at 37 ℃ for 15 min.

For calibration of RBC, 0.5 mL of the above cell suspension was taken in an EP tube, diluted to 5 mL by adding distilled water, shaken for 1 min, and measured at 541 nm with an absorbance value of 0.5 (± 5%). The treated RBC suspension was sealed and stored at 4 °C.

Then the irritation evaluation was performed: the hemolysis rate of different concentrations of samples in the presence of RBC suspensions was determined, and a fitting curve was made to find the half hemolysis rate H50. After that, a single denaturation ability DI was determined, i.e., the critical concentration (i) at which hemolysis of erythrocytes in the presence of samples in the presence of the RBC suspension was observed to undergo a change in colour, and the value of the DI under the action of this concentration was determined. Finally, find the lysis/denaturation ratio (L/D) = H50/DI, and determine the irritation of the sample.

Definition: R1 = (A575 (positive control)—A575 (negative control))/(A540 (positive control)—A540 (negative control)), for oxyhaemoglobin, R1 is approximately 1.05 ± 0.001. (X ± SEM; n = 200).

R2 is the internal standard SDS (concentration of 0.1%) value, which is approximately 0.65; Ri (A) = sample group A575/sample group A540; DI (A) = 100*(R1-Ri)/(R1-R2); L/D = H50/DI.

Based on the DI and H50 obtained from the above calculations and analysed, and used to obtain the L/D values, which corresponded to the data from the acute eye irritation experiments, as shown in Table 2.

**Table 2:** Irritation ratings

score	L/D > 100	10 ≤ L/D < 100	1 ≤ L/D < 10	0.1 ≤ L/D < 1	L/D < 0.1
categorisation	non-irritant	mild	moderately	thrillingly	severe stimulus

Antihemolytic activity: The experiment was divided into three groups: positive.

control group: 250 μL of RBC suspension, 700 μL of PBS and 50 μL of 0.1% SDS were added to the EP tube; sample group: 300, 400, 500, 600, 700 μL of samples were added to the EP tube, and 250 μL of RBC suspension was added to the EP tube and the sample was topped up to 1 mL; sample + SDS control group: 300, 400, 500, 600, 700 μL of samples and 50 μL of 0.1% SDS were added to the EP tube and the sample was topped up to 1 mL. Sample + SDS control group: 300, 400, 500, 600, 700 μL of sample and 50 μL of SDS solution at 0.1% concentration were added to the EP tubes, PBS was added to 750 μL, and then 250 μL of RBC suspension was added to 1 mL, and the EP tubes of the three experimental groups were placed in the shaking table at 150 r/min 37 ℃ for 15 min, 10,000 r/min 1 min and 10,000 r/min 1 min. The EP tubes of the three experimental groups were incubated at 150 r/min for 15 min at 37 ℃, centrifuged at 10,000 r/min for 1 min, and the supernatant was aspirated 200 μL into a 96-well plate, and the absorbance value was measured at 490 nm for calculating the haemolysis rate.

#### Chick embryo chorionic allantoic membrane test

Under the condition of not touching the egg membrane with tweezers, the 9-day incubated chicken embryos were gently broken through the egg shell, and the inner membrane was gently picked with tweezers after moistening the egg membrane with saline to ensure that the vascular membrane was intact. After observing the vascular integrity, 300 μL of positive control solution, negative control solution, and samples were added dropwise to the surface of the allantoic membrane, respectively, and the time of the appearance of vascular haemorrhage, coagulation, and vascular lysis in the allantoic membrane within 5 min was observed, and each sample was tested in parallel with 6 eggs, and the stimulation scoring (IS) was tested by using the reaction time method (Zhao et al. 2021), and the calculation formula is shown below.

$$IS=\frac{\left(301-secH\right)\times 5}{300}+\frac{\left(301-secL\right)\times 7}{300}+\frac{\left(301-secC\right)\times 9}{300}$$

The stimulation scoring (IS) and classifications are shown in Table 3.

**Table 3:** Stimulus scoring and categorisation

IS score	IS < 1	1 ≤ IS < 5	5 ≤ IS < 9	IS ≥ 9
Classification of stimuli	non-irritant	mild irritation	moderate stimulus	Intensity irritation/corrosion

## Results

### Antioxidant efficacy results

#### Analysis of free radical scavenging results

**Fig. 1:**
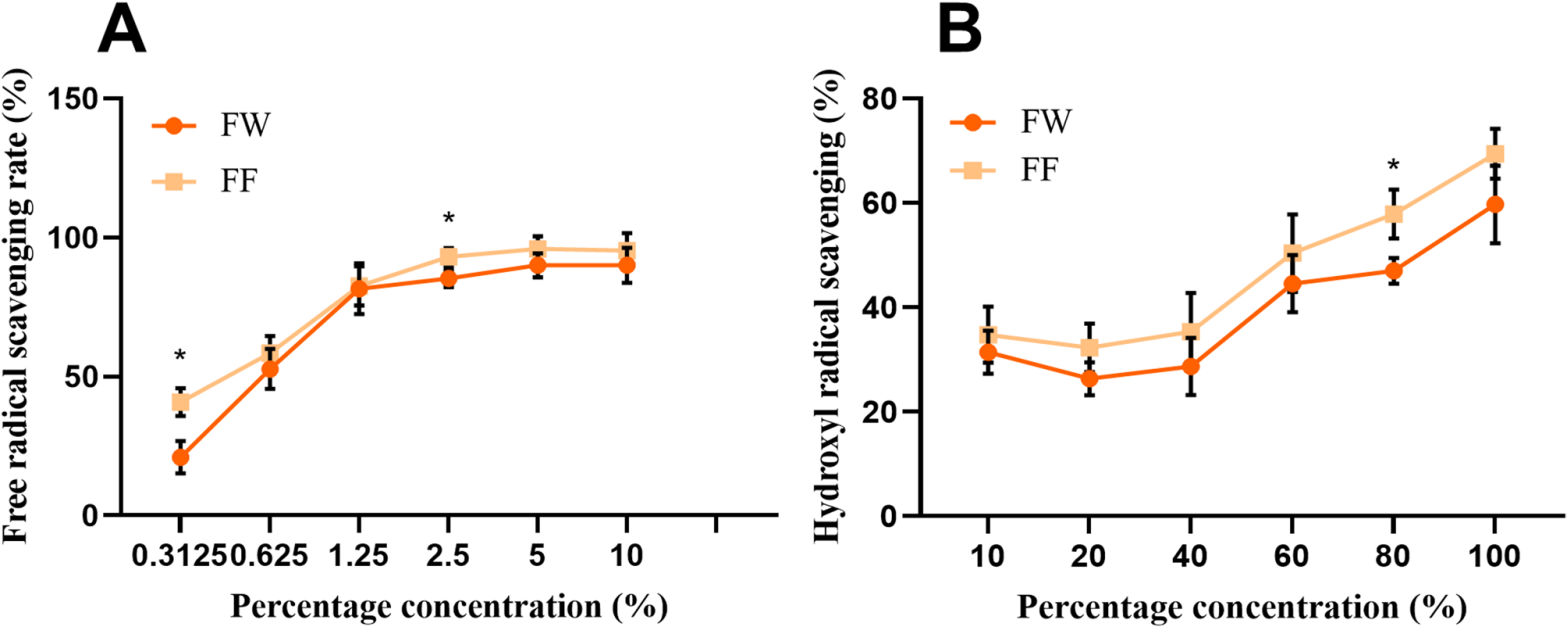
Scavenging of free radicals by FF (**A**: DPPH radicals; **B**: Hydroxyl radicals, Data are presented as mean ± SD (n = 3). *p < 0.05, FF vs FW)

The scavenging effect of the FF on DPPH radicals and hydroxyl radicals is shown in Fig. 1.

Notably, FF and FW showed comparable DPPH and hydroxyl radical scavenging activities in these cell-free assays, As can be seen from Fig. 1, the scavenging effect of both FF and FW on DPPH radicals was very obvious and the scavenging rates of both were similar. The scavenging rate increased exponentially with the increase of the volume fraction of the samples before 5% volume fraction, and then the increase of scavenging rate slowed down with the increase of the volume fraction, and the scavenging rate of the FF reached more than 90% when the volume fraction was 10%. The IC50 for DPPH radical scavenging by FF was 0.525 mg/mL. The IC50 of FW against DPPH radicals was 0.545 mg/mL.

In hydroxyl radical scavenging, both the FF and the FW were effective in scavenging hydroxyl radicals. The scavenging rate grew with the increase of sample volume fraction. The IC50 for hydroxyl radical scavenging by FF was 18.15 mg/mL. The IC50 of FW against hydroxyl radicals was 37.42% 18.71 mg/mL.

The enhanced bioactivities observed in subsequent cellular models are therefore likely attributable to factors beyond mere radical scavenging capacity, such as improved bioavailability or synergistic effects between metabolites. Hydroxyl radical scavenging experiments require higher concentrations, as the scavenging rate does not change significantly at low concentrations (Fig. 1B). Only safe concentrations ≤ 2% are used in cell experiments to avoid toxicity.

#### Analysis of the results of the ABTS method & Determination of reactive oxygen content

**Fig. 2:**
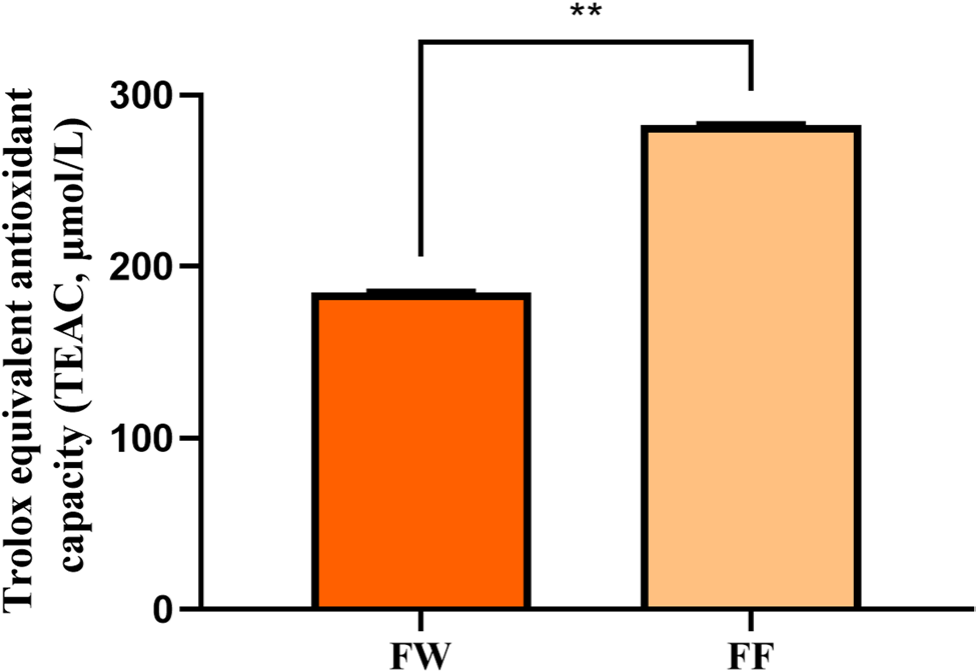
Total antioxidant capacity of FF

Figure 2 shows the total antioxidant capacity of FF as well as FW. From Fig. 2, it can be seen that the total antioxidant capacity of FF was greater than that of FW. The Trolox equivalent antioxidant capacity of sample FF was 282.5 ± 3.9 μM, which was significantly higher than that of FW, 184.8 ± 3.7 μM (**p < 0.01), indicating that *Lactobacillus plantarum* fermentation increased the antioxidant activity of forsythia by 35%.

**Fig. 3:**
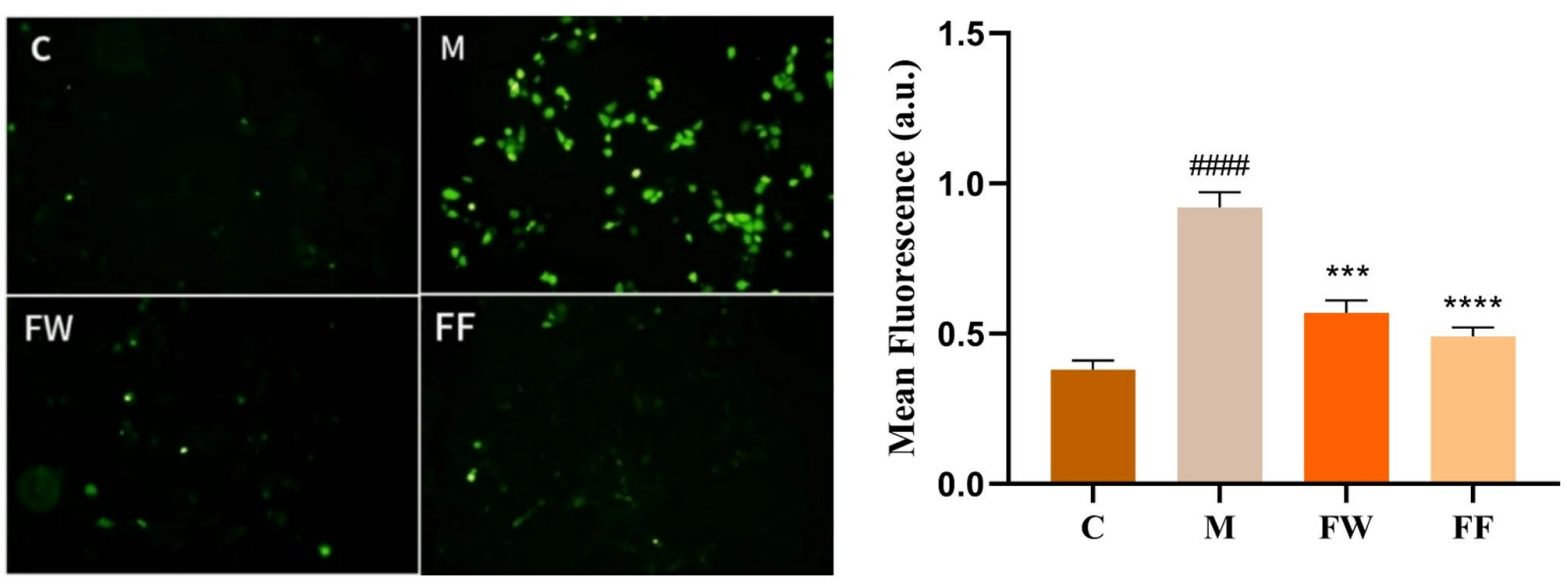
Intracellular ROS generation and quantitative analysis (C: Control group; M: UVB irradiation model group)

ROS generation in HaCaT cells was detected by DCFH-DA fluorescent probe. The scavenging effect of the FF on reactive oxygen species is shown in Fig. 3.

As can be seen from Fig. 3, the blank group (C) had low fluorescence and very low active oxygen content. Quantitative analysis revealed a mean fluorescence intensity of 0.38 ± 0.03 a.u. After UVB irradiation, the content of reactive oxygen species increased significantly to 0.92 ± 0.05 a.u. (####*p* < 0.0001 vs C). Both forsythia FW and fermentation broth treatments demonstrated effective ROS scavenging, reducing fluorescence to 0.57 ± 0.04 a.u. (***p < 0.001) and 0.49 ± 0.03 a.u. (****p < 0.0001) respectively versus UVB-damaged cells. Notably, the FF exhibited superior antioxidant activity, achieving 46.7% greater ROS reduction than the FW.

### Anti-inflammatory & repairing results

#### UVB Dose establishment & HaCaT cell viability

**Fig. 4:**
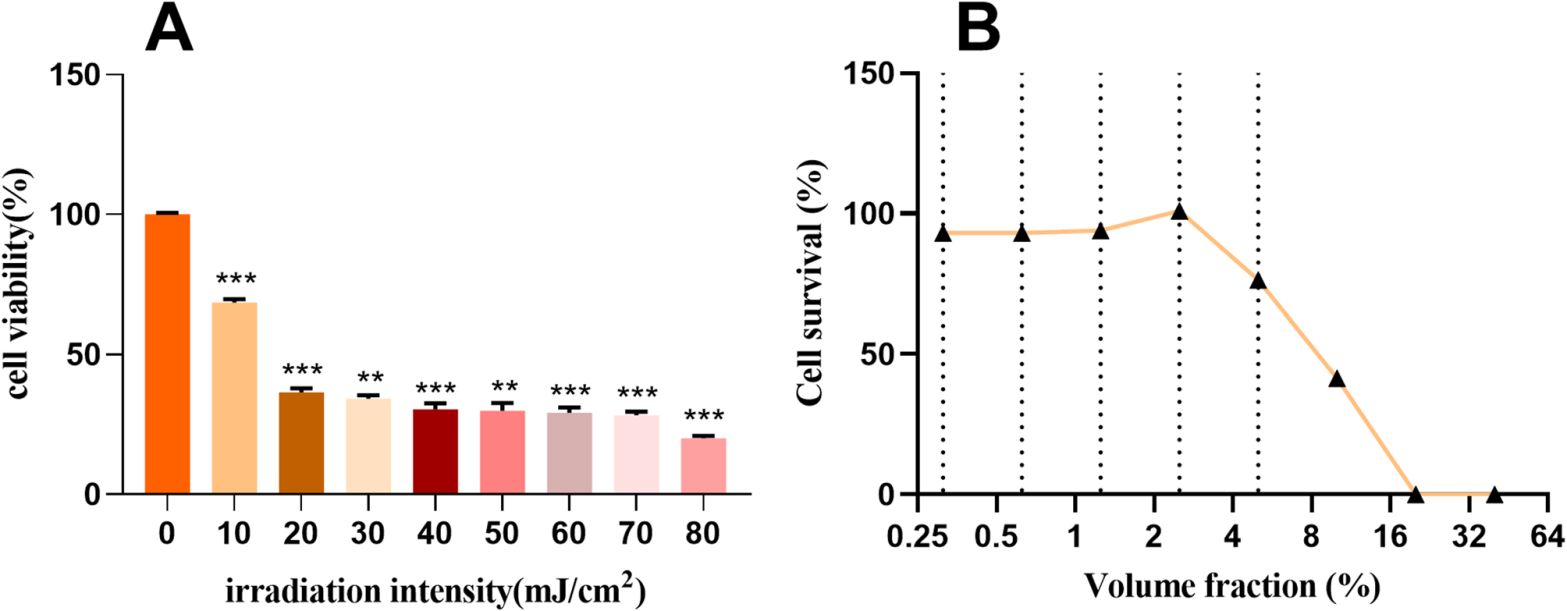
HaCaT cell viability (**A**:Effect of different doses of UVB on the viability of HaCaT cells) (**B**: Effect of different volume fractions on the viability of HaCaT cells)

The results of cytotoxicity after UVB injury using the CCK8 method are shown in Fig. 4. HaCaT cells were damaged using different radiation doses of UVB, and the larger the radiation dose the lower the cell viability, the cell viability was 50% yielding a UVB model with an irradiation intensity of 19.18 mJ/cm^2^. The UVB irradiation intensity was 0.5 mW/cm^2^, and the irradiation time was 38.36 s to achieve a total dose of 19.18 mJ/cm^2^.

The effect of different fermentation broth volume fractions on HaCaT cell survival is shown in Fig. 4 B. the cell viability was better when the volume fraction was 1.25–2.5% and the sample was non-toxic to HaCaT cells, whereas the sample exhibited toxicity to HaCaT cells when the volume fraction was 5%. Therefore, the FF with a volume fraction of 2.0% was selected for subsequent antioxidant efficacy studies, anti-inflammatory efficacy studies, and safety efficacy studies.

#### Inflammatory factor content results

UVB irradiation causes an increase in skin damage and inflammation due to an increase in the level of inflammatory factors secreted by keratinocytes with inflammation, which is one of the representative immune responses to inflammatory stimuli.TNF-α, IL-6, IL-8, and IL-1β are significant inflammatory markers (Sun et al. 2022).

**Fig. 5:**
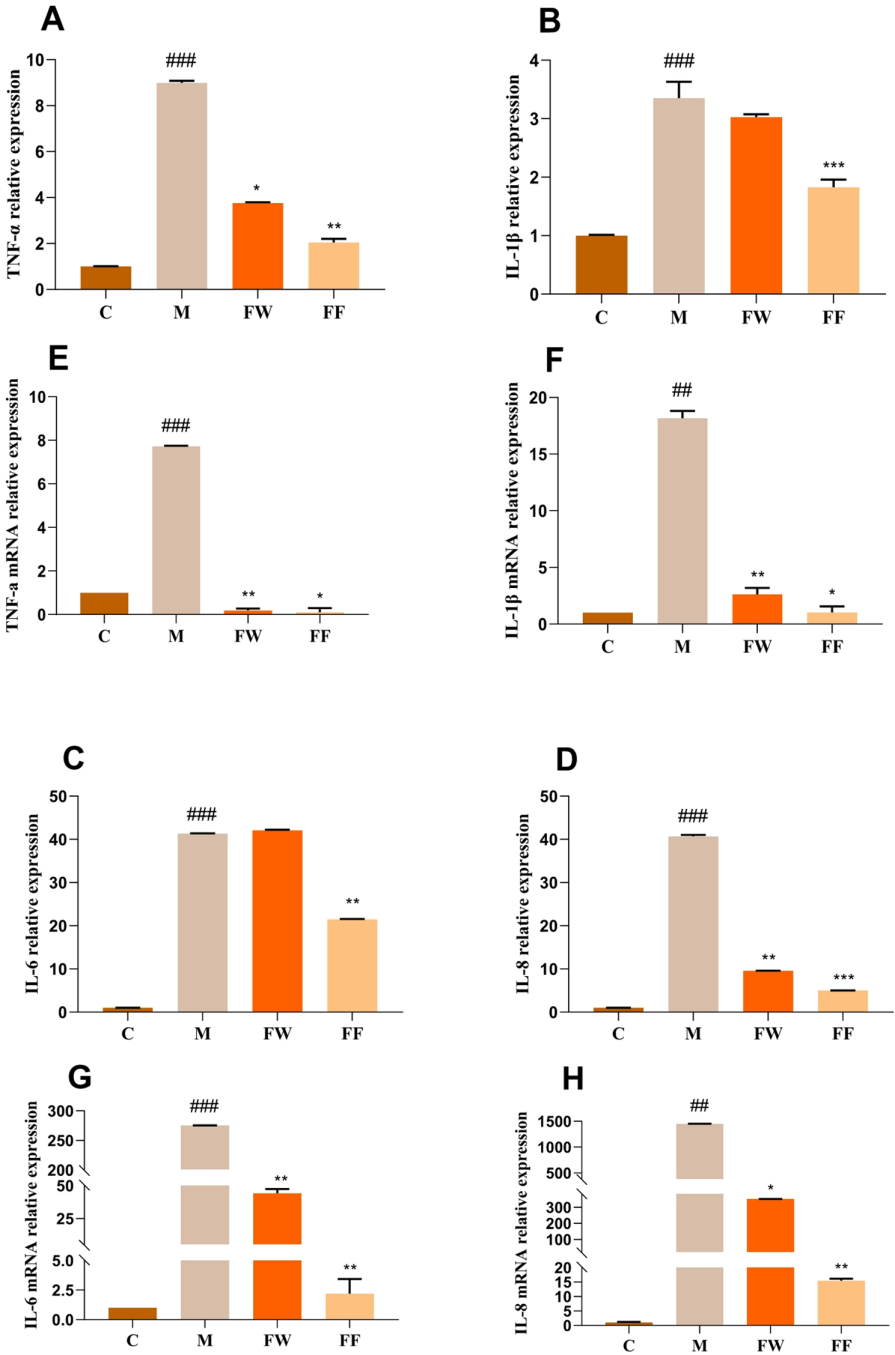
Regulation of UVB-induced cellular inflammatory factor levels by FF (C: Control group; M: UVB irradiation model group) (**A**, **E**) Changes in TNF-α protein and gene levels; (**B**, **F**) Changes in IL-1β protein and gene levels (**C**, **G**) Changes in IL-6 protein and gene level; (**D**, **H**) Changes in IL-8 protein and gene level

Changes in the content of the four inflammatory factors were detected from the protein level using ELISA, and the results are shown in Fig. 5 (A,B,C,D), and the changes in the expression of the four inflammatory factors were detected from the transcriptional level using qRT-PCR in the near future, and the results are shown in Fig. 5 (E,F,G,H).

In terms of protein expression levels, when UVB irradiated HaCaT cells, TNF-α protein content increased ninefold, IL-1β protein content increased 3.4-fold, and IL-6 as well as IL-1β protein content significantly increased 41-fold compared to the blank group. The results indicated that UVB produced inflammatory damage to HaCaT cells. After treating the damaged cells with FW and FF, respectively, FW had an insignificant effect on the reduction of IL-1β and IL-6 protein expression compared to the model group, while FF significantly reduced the expression of four inflammatory factor proteins.

At the level of gene transcription, when UVB irradiated HaCaT cells, compared with the blank group, TNF-α protein content increased 7.7-fold, IL-1β protein content increased 18-fold, IL-6 protein content increased significantly by 275-fold, and IL-1β protein content increased significantly by up to 1450-fold. The results indicated that UVB induced upregulation of inflammatory gene expression in HaCaT cells. After treating the damaged cells with FW and FF, respectively, compared with the model group, FW and FF had a significant reduction effect on IL-6 and IL-8 gene expression, while the regulation of gene expression of TNF-α and IL-1β almost returned to the normal level.

The results showed that FF could reduce the level of inflammatory factors and even tend to normalize the level, and the effect was better than that of FW.

#### Apoptosis factor content results

The onset of apoptosis is inextricably linked to inflammation. Caspase-9 is the initiator of apoptosis and activates effector Caspase-3, Caspase-3 then activates the DNA enzyme to begin the process of causing apoptosis. Bcl-2 can exert an anti-apoptotic effect, and overexpression of Bcl-2 can lead to alteration of redox homeostasis in the nucleus, which reduces caspase activity.Bax, a member of the Bcl-2 family involved in apoptosis, induces apoptosis, and the Bax/Bcl-2 ratio is the initiator switch that initiates apoptosis (Zimmermann and Green 2001; Tang et al. 2019).

**Fig. 6:**
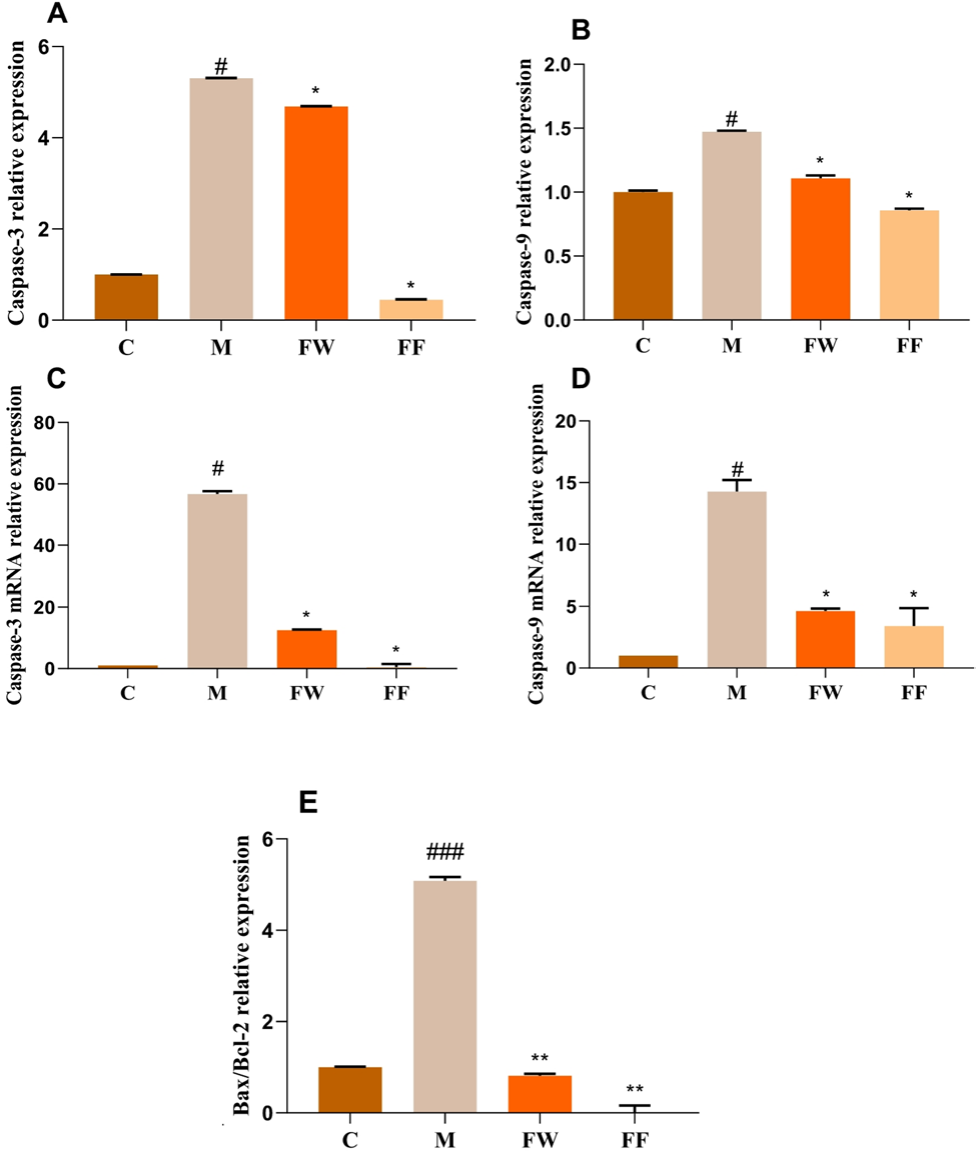
Regulation of UVB-induced apoptotic factor content by FF (C: Control group; M: UVB irradiation model group) (**A**, **C**) Changes in Caspase-3 protein and gene levels; (**B**, **D**) Changes in Caspase-9 protein and gene levels (**E**) Changes in Bax/Bcl-2 ratio values

Changes in the content of two apoptotic factors were detected from the protein level using ELISA, and the results are shown in Fig. 6 (A,B), and the results of the changes in the expression of four apoptotic factors were detected from the transcriptional level using qRT-PCR in the near future, and the results are shown in Fig. 6 (C,D,E).

From the protein expression level, when UVB damaged HaCaT cells, compared with the blank group, the expression of Caspase-3 protein content in the cells was elevated 5.3-fold relative to the blank group, and Caspase-9 protein content was elevated 1.5-fold. After treating the damaged cells with FF, the relative expression of Caspase-3 and Caspase-9 in the cells tended to normalize compared with the model group.

In terms of gene transcription level, the expression of Caspase-3 gene, Caspase-9 gene and Bax/Bcl-2 ratio in HaCaT cells exposed to UVB radiation increased 56.7-fold, 14-fold and fivefold, respectively, compared with the blank group. After the addition of FF and FW respectively, the expression of Caspase-3 and Caspase-9 genes and the Bax/Bcl-2 ratio in the cells decreased significantly compared with the model group, in which the FF restored the expression of Caspase-3 gene and the Bax/Bcl-2 ratio to the normal level.

The results showed that the FF could reduce the content of apoptotic factor and even tend to the normal level, and the effect was higher than that of the blank control FW. From the synthesis of the above protein expression and gene transcription levels, the FF has the ability of inflammatory repair to cell damage.

### HPLC–MS/MS analysis results

The FW and FF contain a variety of biologically active components, including coumarins (e.g., 7-hydroxycoumarin and inulin), lignans (e.g., forsythia lipids), and cinnamic acid and its derivatives (e.g., caffeic acid). The concentrations and ratios of some of these components change significantly during the fermentation process.

**Fig. 7:**
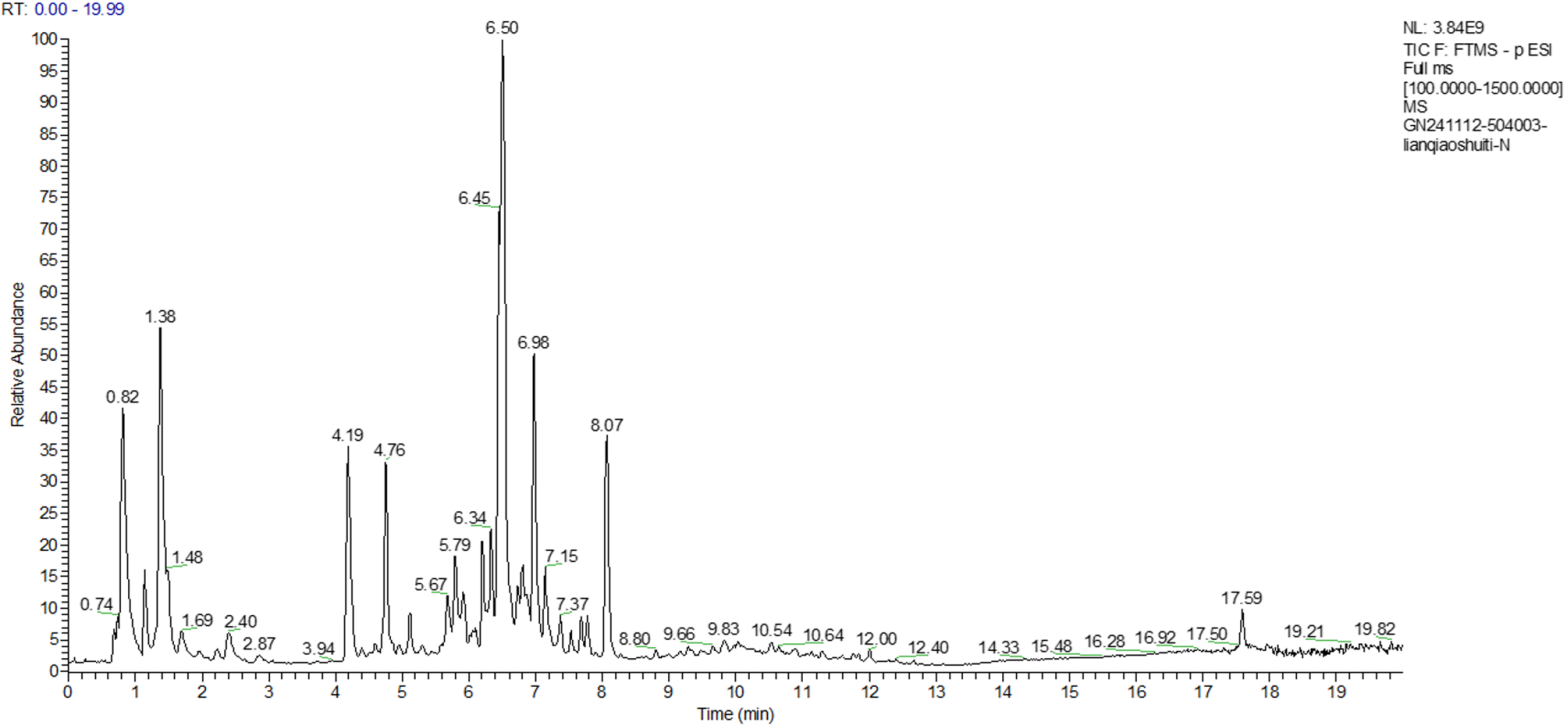
Negative Ion Analysis of FW

**Fig. 8:**
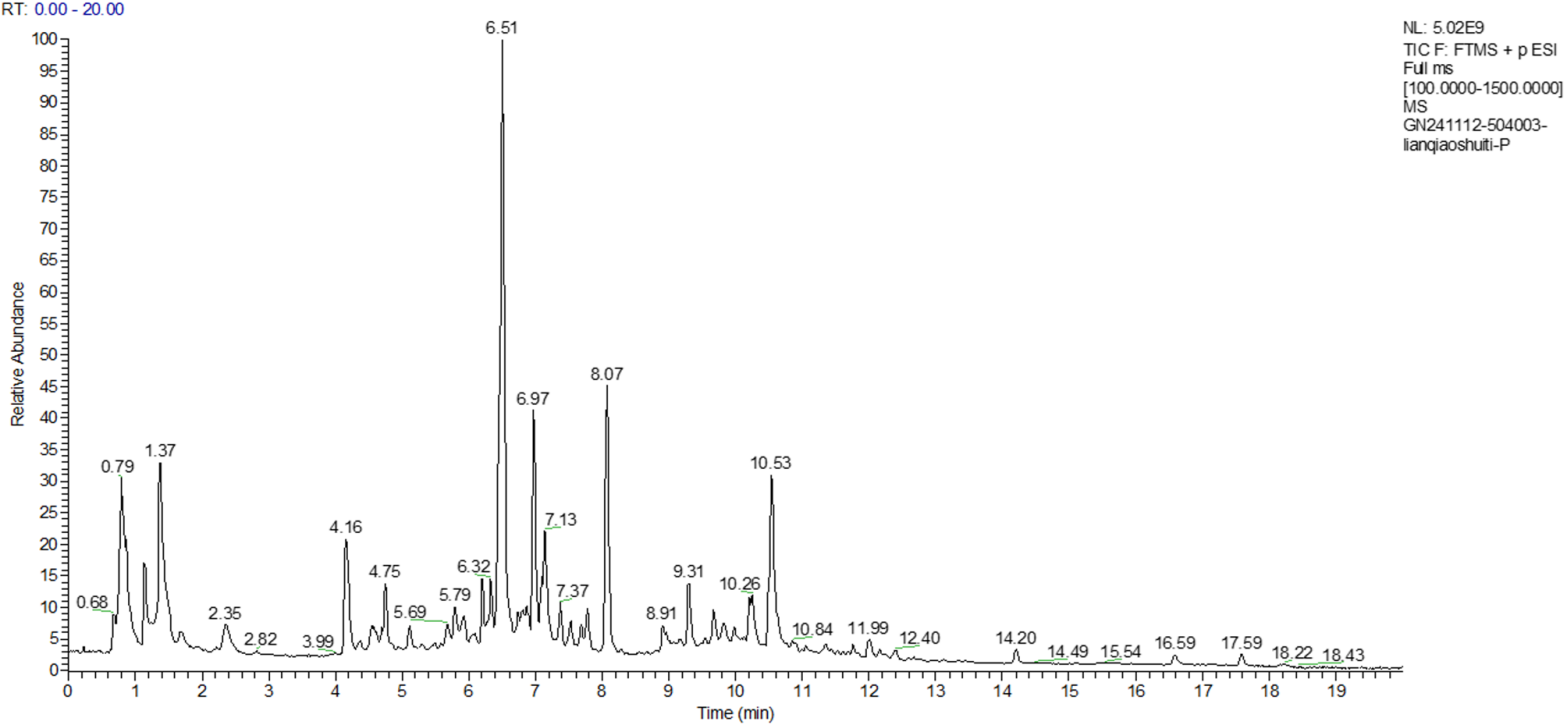
Positive Ion Analysis of FW

**Fig. 9:**
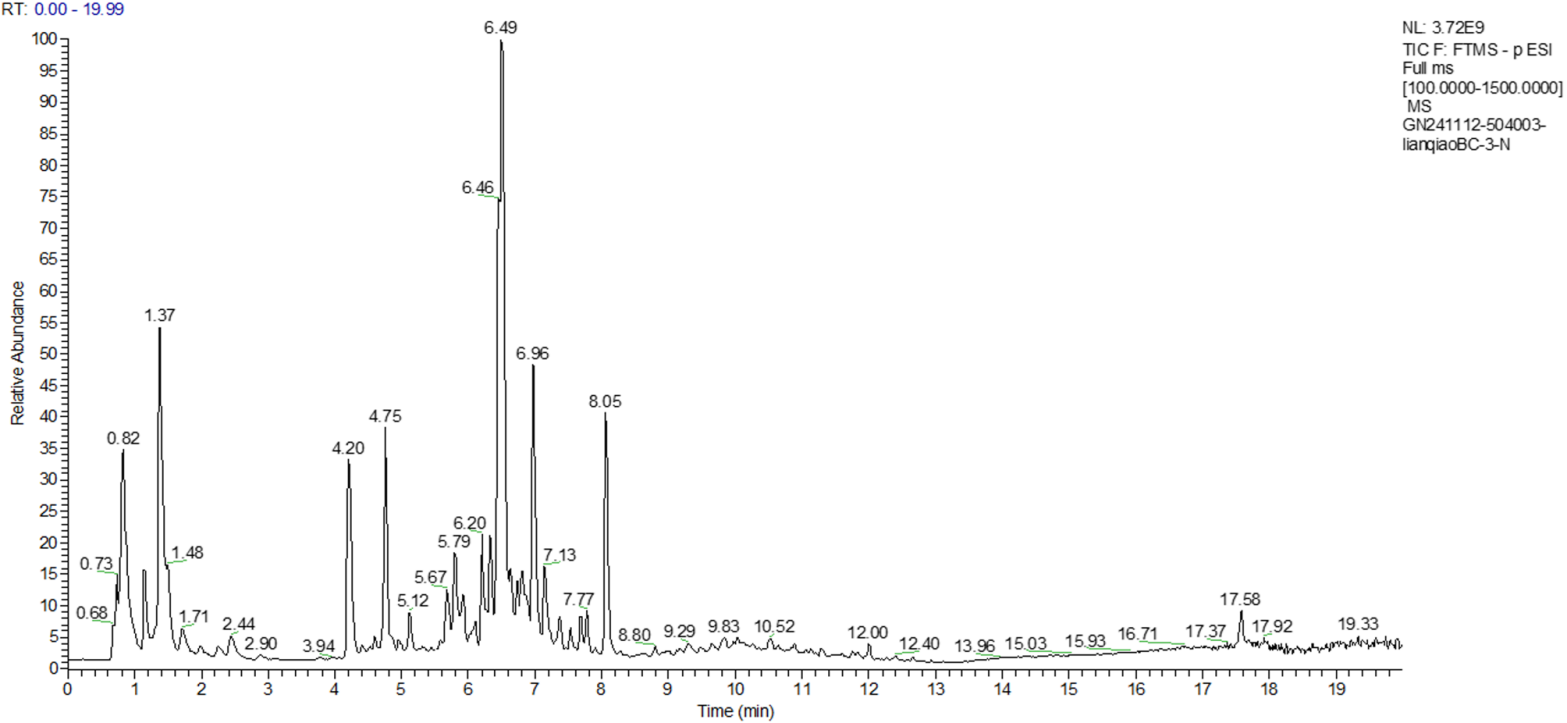
Negative Ion Analysis of FF

**Fig. 10:**
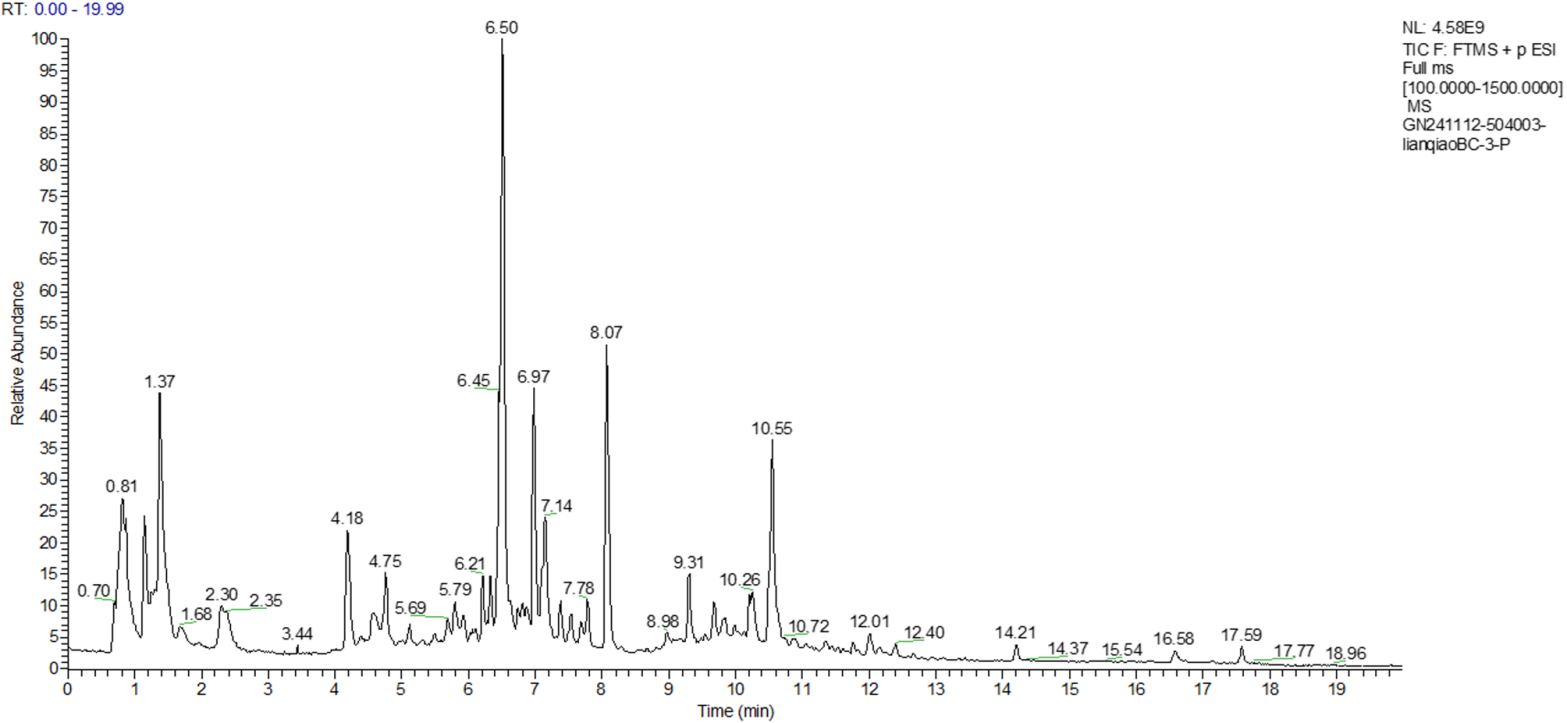
Positive Ion Analysis of FF

Detection of the ion flow diagram (TIC) for each substance are shown in Figs. 7, 8, 9, 10.

The relative concentration of 7-hydroxycoumarins (coumarins) in the fermentation broth, 36.71 μg/mL, was in the highest percentage compared with that in the aqueous extract, 41.01 μg/mL, and the antioxidant activity of the fermentation broth was enhanced by the synergistic effect of metabolites. The relative concentration of forsythia ester glycoside E (organic oxygen compound) in the fermentation broth was elevated by 7.1% (177.92 → 190.54 μg/mL) compared with that in the aqueous extract. The forsythia ester glycosides could reduce the release of pro-inflammatory factors (e.g., TNF-α, IL-6) by inhibiting the NF-κB signaling pathway, which could be directly correlated to the modulation of the inflammatory response of HaCaT cells by the fermentation broth. In addition, the relative percentage of burdock glycosides (lignans glycosides) was elevated by 1.1%, and the fermentation effect may enhance the bioavailability of macromolecules of lignans by partially degrading them, thus strengthening the anti-inflammatory effect.

The concentration of DL-arginine (carboxylic acid derivative) was significantly increased by 35.7% (6.98 → 9.47 μg/mL), which acts as a precursor for nitric oxide NO synthesis and may alleviate UVB-induced ROS accumulation by regulating the redox balance. Meanwhile, the concentration of caffeic acid (cinnamic acid derivative) was increased by 4.3% (60.82 → 63.43 μg/mL), and its catechol structure could efficiently scavenge hydroxyl radicals and superoxide anion radicals.

The results of mass spectrometry analysis indicated that the forsythia fermentation broth enriched anti-inflammatory components such as forsythia ester glycoside E and burdock glycoside, enhanced the antioxidant synergistic effect of DL-arginine and caffeic acid, optimized the bioactivities of lignans components, and significantly enhanced the oxidative stress and anti-inflammatory response capacity of UVB-induced HaCaT cells.

The relative concentrations (μg/mL) of each substance in the progeny before fermentation were specifically shown in Table 4:

**Table 4:** Positive and negative ions analysis results

Name (positive and negative ion analysis)	Classification reference	Relative concentration of each substance in FW	Relative percentage of each substance in FW	Relative concentration of each substance in FF	Relative percentage of each substance in FF
7-Hydroxycoumarin ( +)	Coumarin and derivatives	41.00997984	14.525%	36.70640489	12.139%
Forsythoside E (−)	organic oxygen compound	177.923766	4.961%	190.5449768	5.760%
Arctinoside (−)	lignin glycoside	288.4818611	8.043%	269.0268224	8.133%
forsythia psilocybin ( +)	furanolignan	20.56680576	7.284%	21.31872831	7.050%
forsythia psilocybin (−)	Cinnamic acid and derivatives	60.81714939	1.696%	63.42718756	1.917%
DL-Arginine ( +)	Carboxylic acids and derivatives	6.977659002	2.471%	9.467824044	3.131%
Choline ( +)	organic nitrogen compound	5.573329699	1.974%	6.180360834	2.044%

### Safety efficacy results

#### Erythrocyte hemolysis test results

**Fig. 11:**
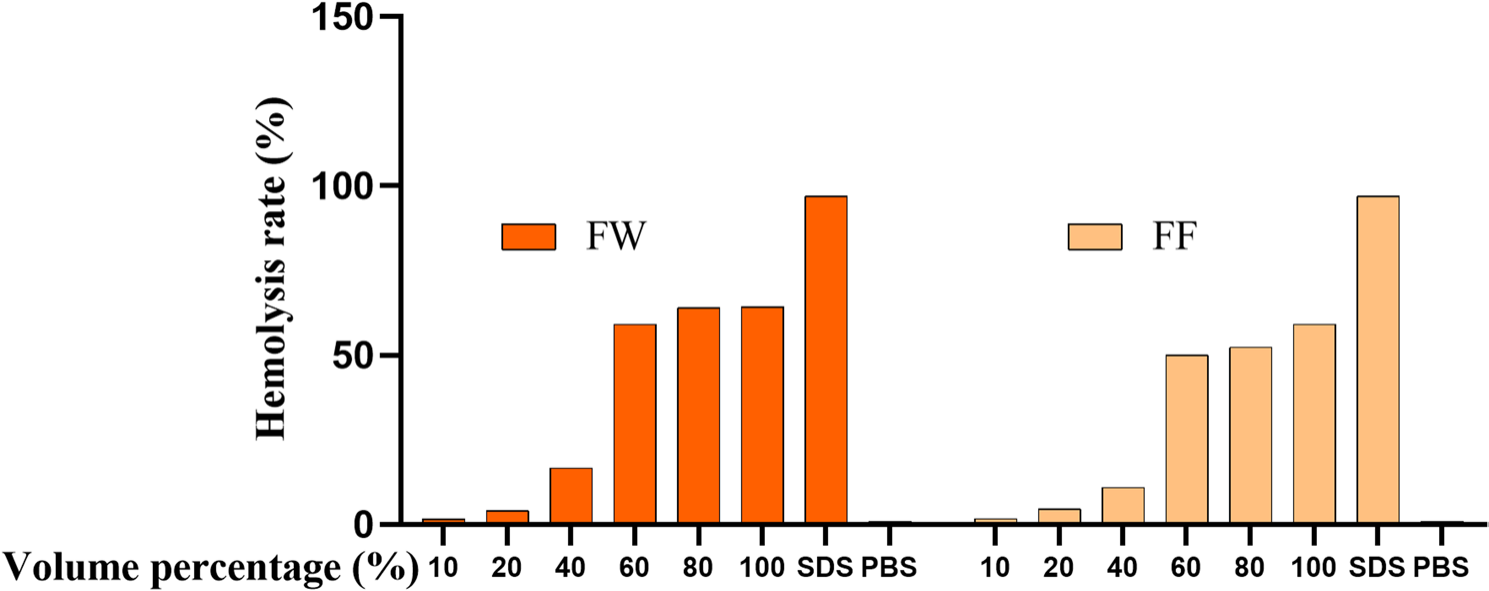
Results of anti-hemolysis assay of FF

Erythrocyte hemolysis assay is used to evaluate the irritation of chemicals on eye tissues by detecting the amount of hemoglobin leakage and the degree of protein denaturation in the erythrocytes (Yu et al. 2020).Fig. 11 shows the results of the anti-hemolysis assay of the FF, which was the hemolysis rate of the erythrocytes of the positive control of 0.1% SDS at the concentration of 0, which was 95.96%. As the concentration of FF increased, the hemolysis rate increased significantly.

According to the categorization in the irritation rating table in Table 5, it can be found that both substances are moderate irritants, in which the L/D value of the fermentation broth is higher than that of the FW, and therefore the irritation of the fermentation broth is lower than that of the FW. Because of the presence of water in the fermentation broth stock solution, it would have some influence on the experimental results, so it was added to the chick embryo chorionic villus allantoic membrane test. The chick embryo chorionic allantoic membrane test (HET-CAM) is a classical in vitro assessment of eye irritation and is suitable for the evaluation of cosmetic products or raw materials (Lin 2020).

**Table 5:** Irritation evaluation

Specimens	H50 (%)	DI (%)	L/D	Evaluation of irritation
FW	74.52	15.79	4.72	moderately irritating
FF	72.17	14.72	4.90	moderately irritating

#### Results of the chick embryo chorionic villus allantoic membrane test

**Fig. 12:**
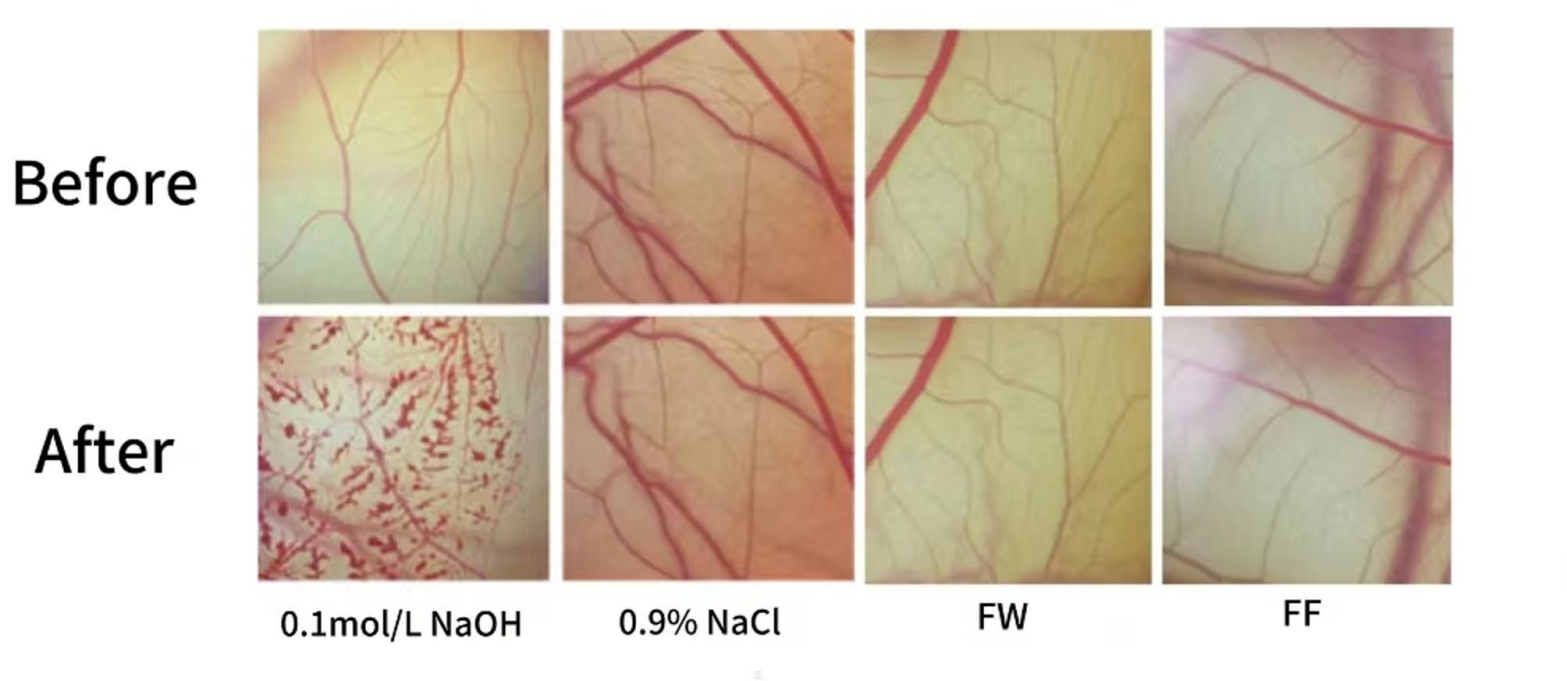
Results of eye irritation measurement of FF

The test results are shown in Fig. 12 using 0.1 moL/L NaOH as the positive control solution, the irritation score was 14.87, the hemolysis of blood vessels was obvious, and it had strong irritation; using saline as the negative control solution, the irritation score was 0.04, and there was no hemolysis of blood vessels; the FW of forsythia and fermentation broth were tested at a volume fraction of 100%, the irritation scores were 0.09, 0.08, and there was no hemolysis of blood vessels The results showed that the FW of forsythia and the fermentation solution were not eye irritants.

## Conclusions

The FW of forsythia was prepared by aqueous extraction method, and the fermentation broth of forsythia was prepared by biofermentation of *Lactobacillus plantarum*. A model of UVB-damaged HaCaT cells was established with a modeling dose of 19.18 mJ/cm^2^. and cell viability was tested for different fermentation broths, and the fermentation broth with a volume fraction of 2% was selected for use in the experiments. The two extracts were evaluated for their efficacy after UVB induction including having antioxidant, anti-inflammatory repair, and safety.

In the study of antioxidant efficacy, the ability of FF to scavenge DPPH radicals, hydroxyl radicals, ABTS radicals and reactive oxygen species was determined at different concentrations. The results showed that the FF had strong scavenging activity against DPPH radicals, hydroxyl radicals, ABTS radicals and reactive oxygen species, and the scavenging rate of the FF against DPPH radicals was gradually enhanced with the increase of the concentration in the range of 0.3%-2.5% v/v. The scavenging rate of the FF against hydroxyl radicals was also enhanced in the range of 10%-100% v/v. The scavenging rate of the FF against ABTS radicals and reactive oxygen species was also strengthened in the range of 0.3%-2.5% v/v. In the range of 10%-100% v/v, the scavenging rate of hydroxyl radicals by the fermentation broth was gradually enhanced with the increase of concentration. From the results of scavenging ABTS radicals and reactive oxygen species, not only the scavenging effect was better, but also the antioxidant effect of the fermentation broth was better than that of the FW.

In the study of cellular inflammatory repair of UVB damage, inflammatory factors (TNF-α, IL-6, IL-8, IL-1β) and apoptotic factors (Caspase-3, Caspase-9, Bax, Bcl-2) in the damaged model cells were used as research indexes, and the protein expression as well as gene transcription were probed by using qRT-PCR and ELISA to investigate the effects of FF was used to investigate the repairing effect of forsythia on damaged cells in terms of protein expression and gene transcription. The experiments showed that the FF could significantly reduce the content of inflammatory factors and apoptotic factors in the damaged cells, and had a better anti-inflammatory and repairing effect, which provided a theoretical basis for the FF to protect the HaCat cells from the inflammatory reaction induced by UVB.

In the study of HPLC–MS/MS analysis, the fermentation process modulated the metabolite profile of forsythia, which was associated with its enhanced efficacy and enhanced the oxidative stress and anti-inflammatory response of UVB-induced HaCaT cells.

In the safety and efficacy study, the FW of forsythia and the original liquid of the fermentation solution were obtained to have moderate irritation by erythrocyte hemolysis test. By adding chicken embryo allantoic membrane eye irritation test, the irritation of forsythia FW and the original solution of the fermentation solution on the blood vessels inside 9-day-old eggs was detected, and the experimental results showed that the FF did not have any significant irritation on the blood vessels. It was verified that the FF was used within the safe range and had no damaging effect on cell membranes.

In this study, it was found that forsythia ferment could protect HaCat cells from UVB-induced photoaging in terms of antioxidant, anti-inflammatory and cellular repair. And the fermentation action enriches a variety of anti-inflammatory, antioxidant beneficial ingredients. This study also provides a theoretical basis for the use of forsythia fermentation as a non-irritating cosmetic ingredient, which can be utilized as an efficacious botanical ingredient with antioxidant, anti-inflammatory, and cellular repair effects.

## Discussions

Our study demonstrates that *Lactobacillus plantarum* fermentation significantly enhances the anti-photoaging efficacy of *Forsythia suspensa* extract. While the direct radical scavenging capacities of FF and FW were comparable in chemical-based assays (Fig. 1), the fermentation broth exhibited markedly superior performance in all cellular models, including ROS reduction, and downregulation of inflammatory and apoptotic markers (Figs. 3, 5, 6). This apparent discrepancy highlights a key insight: the benefit of fermentation may not solely lie in increasing the quantity of antioxidants, but rather in modulating the metabolic profile to improve bioavailability and create synergistic effects that are more effectively perceived by cellular systems.

The HPLC–MS/MS analysis (Table 4) provides clues for this enhanced bioactivity. For instance, the increase in forsythoside E, a known NF-κB inhibitor, aligns with the potent anti-inflammatory effects observed. Similarly, the rise in DL-arginine (a precursor for NO synthesis) and caffeic acid (a potent phenolic acid) could synergistically contribute to regulating the cellular redox environment, potentially activating cytoprotective pathways like Nrf2. Furthermore, microbial fermentation often breaks down larger glycosides into smaller, more bioavailable aglycones, which could explain the significantly stronger cellular effects despite similar in vitro antioxidant readings. Thus, the overall reshaping of the metabolite composition, rather than the dramatic enrichment of a single compound, is likely the driving force behind the superior efficacy of the fermented product.

These findings establish a theoretical foundation for developing forsythia fermentation products as non-irritating cosmetic ingredients with multifunctional dermoprotective effects. While demonstrating significant anti-inflammatory and anti-photoaging efficacy within tested concentrations, further investigations are required to elucidate its comprehensive mechanisms and broader applications.

## Data Availability

The datasets used during the current study are available from the corresponding author on reasonable request.
